# The vulnerability of thalamocortical circuitry to hypoxic-ischemic injury in a mouse model of periventricular leukomalacia

**DOI:** 10.1186/s12868-015-0237-4

**Published:** 2016-01-05

**Authors:** Xiao-Bo Liu, Yan Shen, David E. Pleasure, Wenbin Deng

**Affiliations:** Department of Biochemistry and Molecular Medicine, School of Medicine, University of California, Davis, Sacramento, CA 95817 USA; Center for Neuroscience, School of Medicine, University of California, Davis, Sacramento, CA 95817 USA; Institute for Pediatric Regenerative Medicine, School of Medicine, University of California, Davis, CA 95817 USA; Medical College, Hubei University of Arts and Science, Xiangyang, Hubei China; Department of Biochemistry and Molecular Medicine, School of Medicine, University of California, Davis, 2425 Stockton Blvd., Room 653, Sacramento, CA 95817 USA

**Keywords:** Prematurity, Periventricular leukomalacia, White matter injury, Oligodendrocyte, Thalamocortical circuitry, Cognitive impairment

## Abstract

**Background:**

Periventricular leukomalacia (PVL) is the leading cause of neurological disabilities including motor and cognitive deficits in premature infants. Periventricular leukomalacia is characterized by damage to the white matter in the immature brain, but the mechanisms by which damage to immature white matter results in widespread deficits of cognitive and motor function are unclear. The thalamocortical system is crucial for human consciousness and cognitive functions, and impaired development of the cortico-thalamic projections in the neonatal period is implicated to contribute importantly to abnormalities of cognitive function in children with PVL.

**Results:**

In this study, using a mouse model of PVL, we sought to test the hypothesis that PVL-like injury affects the different components of the thalamocortical circuitry that can be defined by vesicular glutamate transporters 1 and 2 (vGluT1 and vGluT2), both of which are required for glutamatergic synaptic transmission in the central nervous system. We combined immunocytochemistry and immuno-electron microscopy to investigate changes in cortico-thalamic synapses which were specifically identified by vGluT1 immunolabeling. We found that a drastic reduction in the density of vGluT1 labeled profiles in the somatosensory thalamus, with a reduction of 72–74 % in ventroposterior (VP) nucleus and a reduction of 42–82 % in thalamic reticular nucleus (RTN) in the ipsilateral side of PVL mice. We further examined these terminals at the electron microscopic level and revealed onefold–twofold decrease in the sizes of vGluT1 labeled corticothalamic terminals in VP and RTN. The present study provides anatomical and ultrastructural evidence to elucidate the cellular mechanisms underlying alteration of thalamic circuitry in a mouse model of PVL, and reveals that PVL-like injury has a direct impact on the corticothalamic projection system.

**Conclusions:**

Our findings provide the first set of evidence showing that the thalamocortical circuitry is affected and vulnerable in PVL mice, supporting a working model in which vGluT1 defined corticothalamic synapses are altered in PVL mice, and vGluT2 defined thalamocortical synapses are associated with such changes, leading to the compromised thalamocortical circuitry in the PVL mice. Our study demonstrates that the thalamocortical circuitry is highly vulnerable to hypoxia–ischemia in the PVL model, thus identifying a novel target site in PVL pathology.

## Background

Periventricular leukomalacia (PVL) is the predominant brain pathology in premature infants and is the leading cause of subsequent neurological disability including spastic motor deficits (cerebral palsy) and cognitive impairments. Nearly 90 % of the 13–15 million premature infants born worldwide every year survive beyond infancy, in which ≈5–10 % of the survivors develop cerebral palsy, and 40–50 % develop cognitive and behavioral deficits [3, 25]. Periventricular leukomalacia is traditionally classified as a white matter disorder. However, white matter damage underlying PVL is now recognized as the major component of a more generalized injury to the cerebrum that includes neuronal and axonal injury, and is renamed “encephalopathy of prematurity” [25]. Emerging neuropathological and neuroimaging studies demonstrate that PVL is associated with injury to many cortical and subcortical regions, including thalamus, cerebral cortex, hippocampus and basal ganglia in variable combinations [4, 11, 21, 25]. The widespread effect of the disorder may account for cognitive impairments and intellectual deficits in preterm survivors with PVL. In particular, white matter injury impacts on axon fibers ascending and descending from cerebral cortex and thalamus and the reciprocal thalamo-cortical circuitry that is crucial for generating and maintaining awakening-sleeping cycle of high mammals and thalamocortical oscillations that are essential for human consciousness and cognitive function.

It has been widely accepted that thalamocortical connections are formed perinatally in the mouse, and the synaptic remodeling and circuitry refinement start around P7 and continue in the second postnatal week [1, 7, 20]. The functional thalamocortical circuitry is established around P15 [12]. The second postnatal week (P7–P15) is critical for establishing thalamic oscillations that are crucial for the maturation of the thalamocortical circuitry. Using a mouse model of PVL [23, 24] we investigate whether PVL induction affects the establishment of the thalamocortical circuitry and whether synaptic connections in the thalamocortical circuitry are altered following PVL induction. White matter injury in PVL may affect a significant portion of glutamatergic axon terminals connecting the somatosensory thalamus and the cerebral cortex. Defects in these axon terminals can be identified at cellular and ultrastructural level using specific glutamatergic synaptic markers, such as vGluT1 and vGluT2, which have been used to define specific thalamo-cortical systems [9, 13]. Gene expression and immunocytochemistry studies have confirmed that vGluT1 is primarily expressed in layer III and layer V-VI pyramidal cells and vGluT2 is highly expressed in thalamic relay cells and this complementary expression is reflected more clearly in the protein expressions that mainly occur in the axon terminals. It has been previously shown that vGluT1 specifically labels the corticothalamic synapses, whereas vGluT2 specifically defines the thalamocortical synapses [9, 13]. We demonstrated that the synaptic contacts between vGluT2-labeled thalamocortical axon terminals and NG2 + oligodendroglial progenitor cells (OPCs) in the developing white matter were selectively affected and caused the postsynaptic profiles of NG2 progenitor cells to shrink [23]. A recent study further confirmed that these vGluT2 synapses also target NG2 cells in layer III-IV and layer VI [19] that contains pyramidal cell projections as corticothalamic inputs to the somatosensory thalamus. In the present study, we sought to test the hypothesis that PVL-like injury first affects the vGluT2-containing thalamocortical projection system that in turn profoundly compromises vGluT1 containing corticothalamic projection system and thus alters vGluT1 defined corticothalamic synapses in the thalamus. We applied vGluT1 immunocytochemistry and electron microscopy (EM) to analyze the contralateral (control) and ipsilateral (injured) side of the brains of PVL mice. We found that the density of vGluT1 immunolabeled terminals was significantly decreased in the somatosensory thalamus of the ipsilateral injured side. Furthermore, at the EM level, these vGluT1 labeled corticothalamic terminals were drastically altered in their sizes and synaptic structures. These findings provide the first set of evidence showing that the thalamocortical circuitry is affected and vulnerable in PVL mice, supporting a working model in which vGluT1 defined corticothalamic synapses are selectively altered in PVL mice, and vGluT2 defined thalamocortical synapses are associated with such changes, leading to the compromised thalamocortical circuitry in the PVL mice.

## Methods

### Animals

C57B/6 mice (The Jackson Laboratories, Bar Harbor, ME) at postnatal day 6 (P6) were used to characterize neuropathological features under different treatments. NG2-DsRed transgenic mice [The Jackson Laboratories, stock#: Tg(Cspg4-DsRed.T1)1Akik/J] were used to study the identity of postsynaptic profiles in cerebral white matter. Animal research was approved by the University of California-Davis Committee on Animal Research. All procedures were carried out by following the guidelines set by the institutional animal care and use committee.

### Unilateral carotid ligation (UCL) plus hypoxia

Twenty animals underwent a permanent ligation of the right common carotid artery under ice anesthesia, followed by 1-hour recovery on a thermal blanket, maintaining body temperature at 33–34 °C and with a 1-hour feeding period with the dam. Animals were then placed in a sealed chamber infused with nitrogen to maintain a level of 6.0 % O_2_. Different durations of hypoxia were applied. The body temperature of mice was maintained at 33–34 °C by leaving animals on thermal blanket throughout hypoxia. After a 1-hour period of recovery, mice were returned to their dam.

### Immuno-electron microscopy

Ten P10 mice that were subjected to UCL/hypoxia at P6 were perfused with 4 % paraformaldehyde plus 0.2 % glutaraldehyde in 0.1 M phosphate buffer saline (PB, and brains were cut coronally with a vibratome (Leica) at 60 μm. Sections were stained for vGluT1 and vGluT2 antibodies (Millipore, Billerica, MA) using the immunoperoxidase ABC method. A total three cases showing dense vGlut1 immunostaining were selected for electron microscopic study. The stained sections were processed for EM as described previously [9]. The sections from the ipsilateral (injury) side and the contralateral (control) side were first examined in the light microscope at 10X and 20X, the forebrain containing the cerebral cortex and thalamus was identified and photographed, ventroposterior nucleus (VP) and thalamic reticular nucleus (RTN) regions (approximately 2 × 0.5 mm) were cut under a dissecting microscope and glued to blank resin blocks. Thin sections were cut at 70 nm using an ultramicrotome (Leica Ultracut) and collected on single-slot (2 × 1 mm) copper grids and stained with uranyl acetate and lead citrate, and examined in a Philips CM120 EM at 80 kV. The regions were carefully scanned to exclude the areas containing fiber tract in the internal capsule, and the orientation of the region was guided by light microscopic images taken before EM processing. Much effort was made to ensure that the regions were taken from the somatosensory thalamus. Images were taken by a 2 × 2 K CCD camera (Gatan, Inc., Pleasanton, CA) and processed by using the software provided by Gatan, Inc (DigitalMicrograph). Images were composed using Photoshop CS (Adobe System, Mountain View, CA).

### Quantification by light microscopy

vGluT1 immunoperoxidase labeled sections embedded with Araldite were used for the quantitative analysis. For each case (the contralateral and ipsilateral pair), at least one section from each side was examined under light microscope using X20 and X40 objectives. Light microscopic images (X40) showing dense vGluT1 labeled boutons in thalamus (VP and RTN) were captured using a Zeiss imaging system with a CCD camera (Zeiss, Thornwood, NY, USA) and the images were processed in Photoshop CS to adjust the brightness and contrast and convert to gray scale and imported to Image J (NIH, Bethesda, MD, USA) as TIF files. The “analyze particles” function was used to count the labeled profiles. Assuming the diameters of the labeled corticothalamic terminals were from 0.3 to 0.6 μm [17], accordingly, the profile sizes (areas) in a range between 0.07 and 0.7 μm^2^ were included in counting. This range would cover the sizes of most vGluT1 labeled profiles in thalamus. In each section, at least two regions (up to five regions) and each rectangular region approximately 100 μm^2^ were subjected to particle analysis. The particle numbers in each region were obtained, for each case, mean numbers of profiles ± standard deviation/100 μm^2^ was compared in each pair (ipsilateral and contralateral) in VP or RTN, respectively.

### Quantification by electron microscopy

Electron microscope of vGluT1 immunoperoxidase labeled terminals or synapses were taken at X10,000. Electron microscope images were obtained from the contralateral and injured side of VP and RTN. In each side, at least 10–15 images were taken. Electron microscope images were converted to TIF files and imported to Image J for measuring. We used the “Analyze” function to measure the area (in μm^2^) and perimeter (in μm) of each vGluT1 labeled terminals. Given the irregular shape of the labeled profiles and the fact that the area of the profile represents closely its size, we therefore used the area for quantification. At least two labeled terminals were identified from each image and measured. The mean area size (μm^2^) ± standard deviation (SD) from the contralateral or ipsilateral side was compared.

## Results

### Decrease in number of vGluT1 immunolabeled profiles in the somatosensory thalamus of PVL mice

**Fig. 1:**
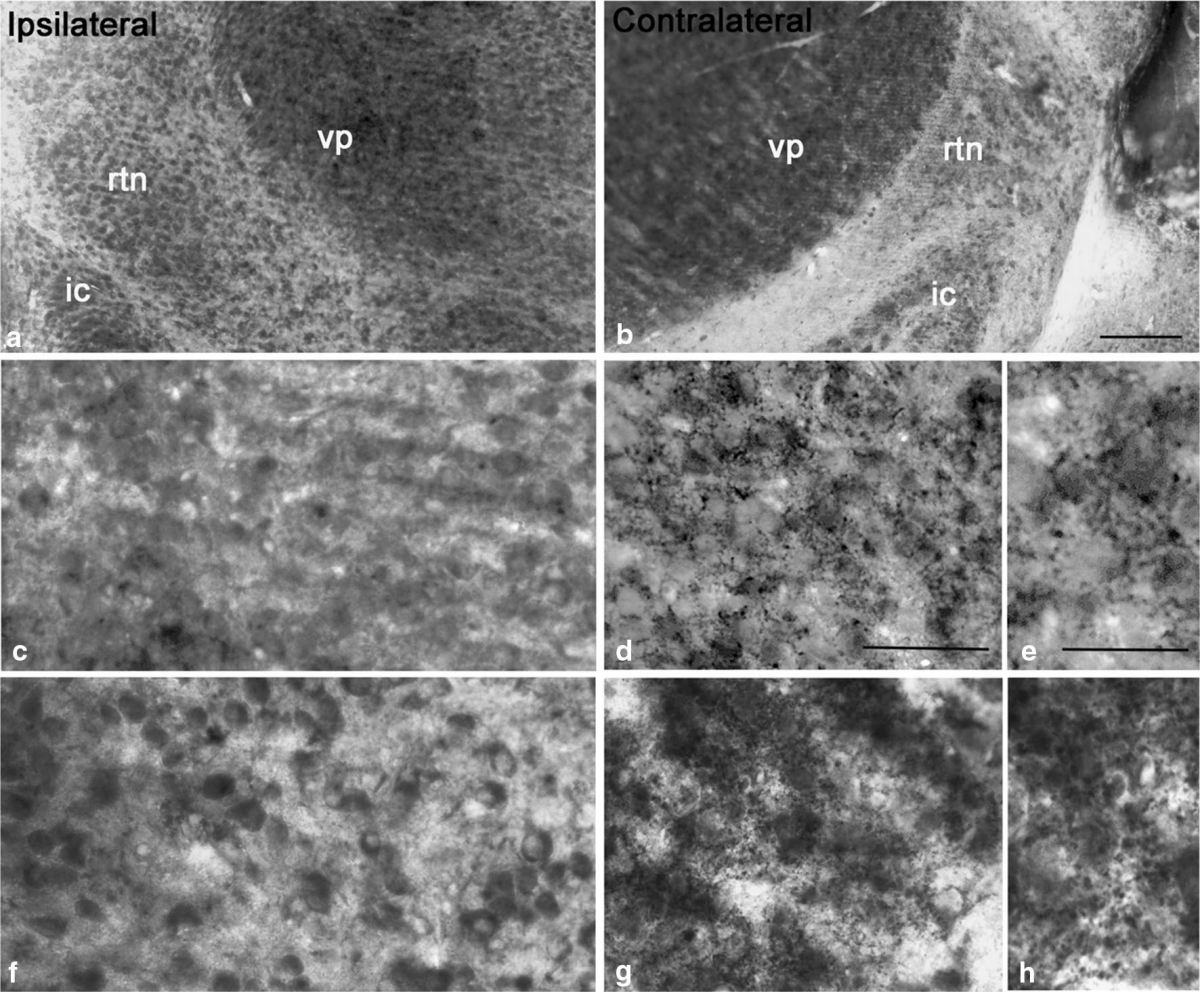
Light microscopic images taken from Araldite embedded vibratome sections showing immunolabeling of vGluT1 in the somatosensory thalamus [ventroposterior (VP) nucleus and thalamic reticular nucleus (RTN)] of ipsilateral (**a**) and contralateral (**b**) sides of the brain in a mouse model of PVL. **c** is a high magnification image taken from VP showing the area lacking vGluT1 labeling in the ipsilateral side. **d** is a high magnification image taken from VP of the contralateral side showing clusters of vGluT1 labeled profiles among neuropils. **e** is a much higher power image showing the labeled profiles including presynaptic boutons. **f** Light microscopic image showing the lack of vGluT1 labeling in the RTN region of the ipsilateral side; note some nonspecifically labeled somata in the region. **g** Light microscopic image showing vGluT1 labeled small boutons and profiles in the RTN of the contralateral side. **h** A high power light microscopic image showing clusters of vGluT1 labeled profiles in the RTN

**Fig. 2:**
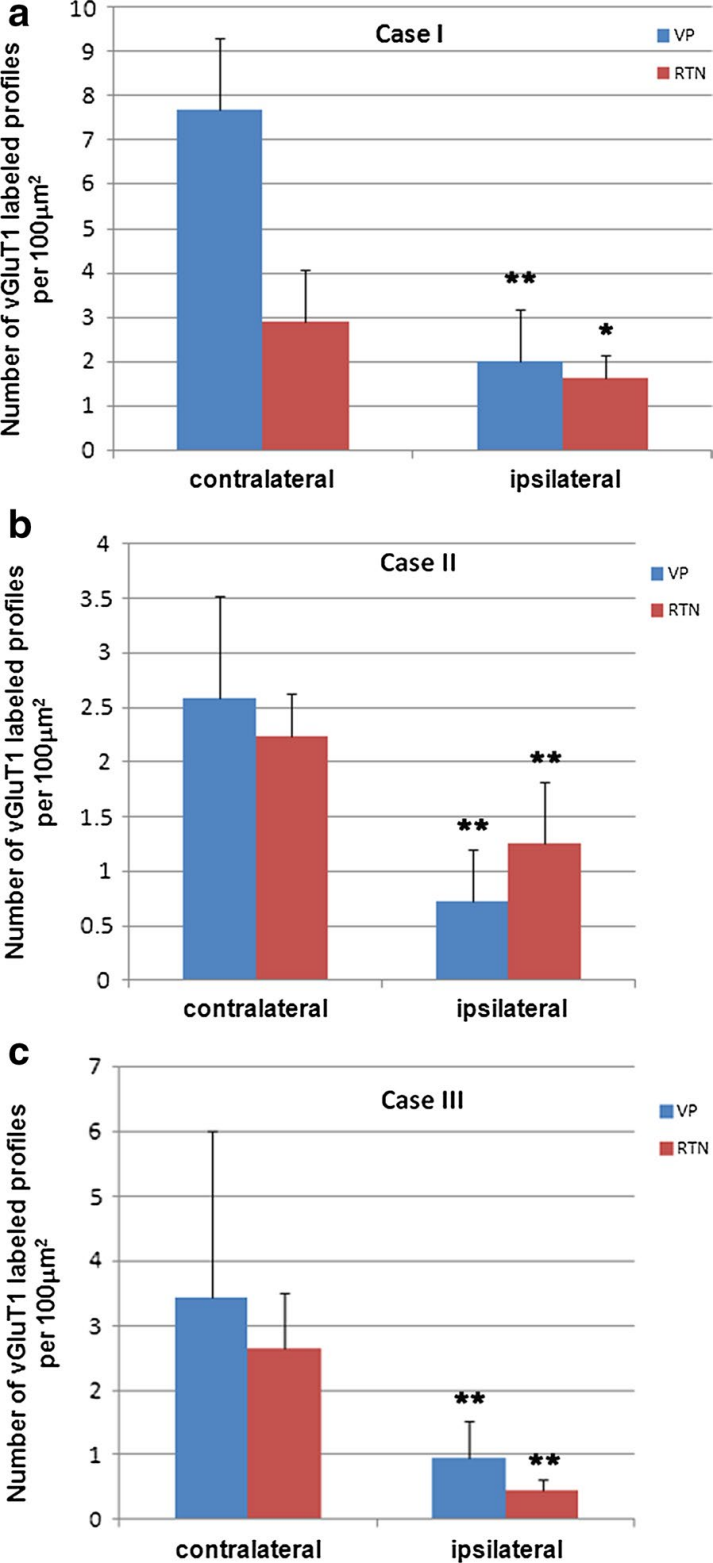
Quantitative analysis of the density (number of vGluT1 labeled profiles per 100 μm^2^) of vGluT1 labeled profiles in the contralateral and ipsilateral side VP (*blue color*) and RTN (*red color*) from three cases of PVL mice. In the contralateral side, the density of vGluT1 labeling is higher in VP than RTN in three cases. In the ipsilateral side, the density is decreased significantly in VP (average 3.8-fold decrease) and RTN (onefold–fivefold decrease) compared to the contralateral side, *p < 0.05; **p < 0.01

At the light microscopic level, vGluT1 immunolabeled profiles appeared to be thin presynaptic terminals and small boutons densely distributed in the dorsal thalamus including VP and RTN of the contralateral side (Fig. 1). In the ipsilateral side, a drastic decrease in the number of vGluT1 immunolabeled profiles was noted in some regions of the dorsal thalamic nuclei including VP and RTN (Fig. 1). Compared to the contralateral side thalamus which contains dense clusters of vGluT1 labeled profiles, the labeling in the ipsilateral side was more diffuse and became less intense in clustering. Instead, some non-specific lightly labeled somata were uniformly distributed on the background, but they were easily differentiated from the labeled terminals by sizes. A quantitative analysis was made to access the change in number of vGluT1 immunolabeled profiles in 100 μm^2^ area of VP and RTN of the ipsilateral side of the somatosensory thalamus (Fig. 2). A total of three independent cases were analyzed and quantified, and a drastic change in vGluT1 labeling density was found in both VP and RTN of the ipsilateral side. In VP, in case 1, the density of labeling was decreased from 7.69 ± 1.61 profiles per 100 μm^2^ (contralateral side) to 2.0 ± 1.17 profiles per 100 μm^2^ (ipsilateral side) and approximately 74 % or 3.8-fold reduction, p < 0.005; in case 2, it was decreased from 2.59 ± 0.93 profiles per 100 μm^2^ (contralateral side) to 0.73 ± 0.47 profiles per 100 μm^2^ (ipsilateral side) approximately 72 % or 3.5-fold reduction, p < 0.0007; in case 3, it was decreased from 3.44 ± 2.58 profiles per 100 μm^2^ (contralateral side) to 0.94 ± 0.59 profiles per 100 μm^2^ (ipsilateral side) approximately 72 % or threefold reduction, p < 0.00216. Consistent with changes in VP, in RTN, in case 1, the density of labeling was decreased from 2.90 ± 1.17 profiles per 100 μm^2^ (contralateral side) to 1.63 ± 0.51 profiles per 100 μm^2^ (ipsilateral side) approximately 44 % or 1.8-fold reduction, p < 0.03; in case 2, it was decreased from 2.24 ± 0.39 profiles per 100 μm^2^ (contralateral side) to 1.26 ± 0.56 profiles per 100 μm^2^ (ipsilateral side) approximately 43 % or 1.8-fold reduction, p < 0.0129; in case 3, it was decreased from 2.64 ± 0.87 profiles per 100 μm^2^ (contralateral side) to 0.46 ± 0.15 profiles per 300 μm^2^ (ipsilateral side) approximately 82 % or fivefold reduction, p < 0.0006 (Fig. 2). Overall, the decreases of vGluT1 labeling density in three cases were consistent in VP and RTN and the differences between contralateral and the ipsilateral sides were highly significant.

### Alteration in ultrastructure of vGluT1 labeled corticothalamic synapses in the somatosensory thalamus of PVL mice

**Fig. 3:**
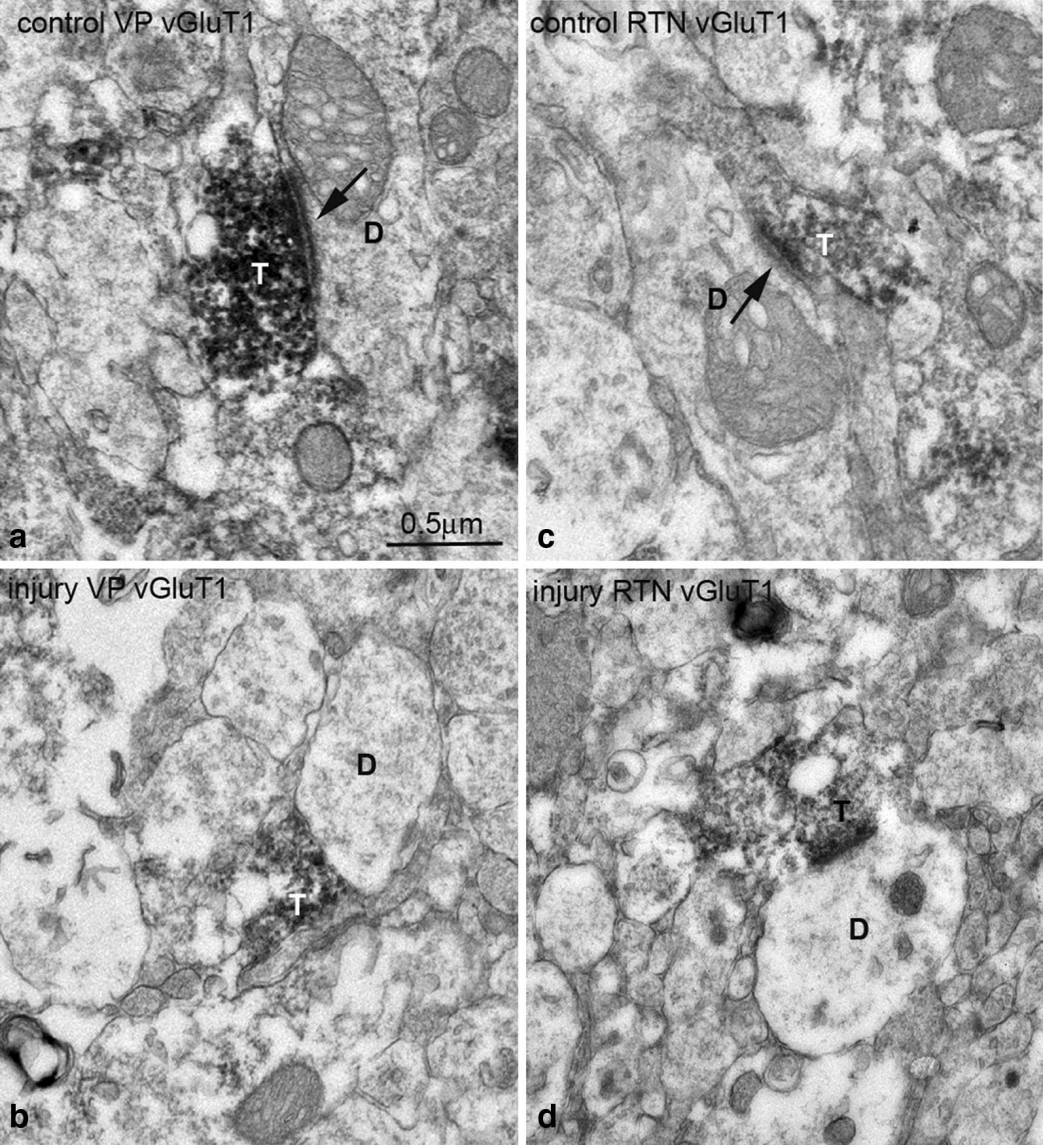
Electron micrographs showing typical vGluT1 immunoperoxidase labeled terminals in the contralateral side VP (**a**) and RTN (**c**) and ipsilateral side VP (**b**) and RTN (**d**). In the contralateral side, vGluT1 labeled terminals (*T*) contain densely packed vesicles and form clear asymmetrical synapses (indicated by *arrows*) with postsynaptic dendrites (*D*) (note the prominent postsynaptic densities associated with the synapses). In ipsilateral side, the labeled terminals have less defined irregular shapes and form less clear synaptic contacts with postsynaptic dendrites (*D*). *Scale bar* = 0.5 μm

**Fig. 4:**
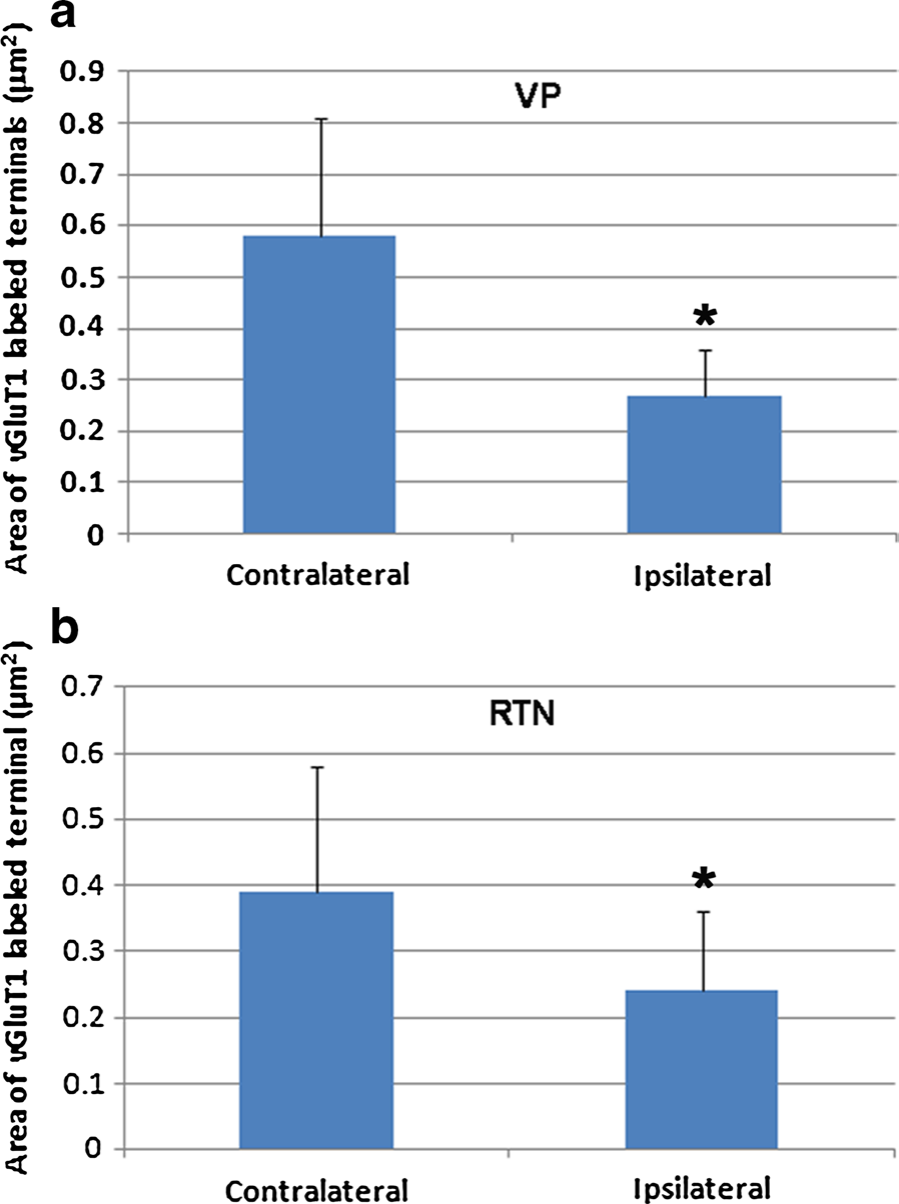
Quantitative analysis of the size (μm^2^) of vGluT1 immunolabeled axon terminals in the contralateral and ipsilateral side of VP (**a**) and RTN (**b**) of PVL mice. In VP, twofold reduction in the size of the labeled terminals is observed in the ipsilateral side (*p < 0.00011). In RTN, 1.6-fold reduction in the size of the labeled terminals is found in the ipsilateral side (*p < 0.0291)

The drastic reduction in number of vGluT1 labeled terminals in the somatosensory thalamus of PVL mice strongly indicated that these terminals and their synapses may undergo major changes in their ultrastructure in PVL mice. We further carried out electron microscopic analysis to resolve this issue. At the EM level, in contralateral side, vGluT1 immunolabeled terminals displayed typical ultrastructural features of corticothalamic terminals in VP and RTN as reported previously [17]: these terminals were about 0.3–0.6 μm in diameter, containing few mitochondria, filled with round or oval shape synaptic vesicles and they usually form asymmetrical synapses associated with prominent postsynaptic densities on dendritic profiles. Interestingly, these terminals usually contact small and presumably distal dendrites of relay cells in VP and contact much wide range sizes of dendritic profiles in RTN [15, 17] (Fig. 3). In contrast to vGluT1 labeled terminals in the contralateral side, the labeled terminals in the ipsilateral side were much smaller in their sizes and they showed some structural alterations including less defined membranes and containing fewer synaptic vesicles and were lack of prominent postsynaptic densities (Fig. 3). To quantitatively access the change in ultrastructure of vGluT1 labeled terminals in the ipsilateral side, a total of 15 images each containing at least two labeled terminals from either VP or RTN of the contralateral and ipsilateral side of PVL mice were subjected to quantitative analysis. In the contralateral VP, the mean area size of vGluT1 labeled terminals was 0.58 ± 0.23 μm^2^, in the ipsilateral side, the mean area size was 0.27 ± 0.09 μm^2^, there was more than twofold decreased in the size of the terminals, the change was highly significant (p < 0.00011). In the contralateral RTN, the mean area size was 0.39 ± 0.19 μm^2^, in the ipsilateral side, the mean size of vGluT1 labeled terminals was 0.24 ± 0.12 μm^2^, there was more than 1.5-fold reduction in the size of these terminals and the change was significant (p < 0.0291) (Fig. 4). We also observed a change in the length of postsynaptic density of vGluT1 labeled synapses in the ipsilateral side compared to the contralateral side.

## Discussion

In the present study, we combined immunocytochemistry and EM to investigate the changes associated with corticothalamic synapses which were specifically identified by vGluT1 labeling in a mouse model of PVL. We found that a drastic reduction in number of vGluT1 labeled profiles in the somatosensory thalamus (VP: a reduction of 72–74 % or about threefold–fourfold; RTN: a reduction of 42–82 % or 1.8-fold–fivefold) in the ipsilateral side of PVL mice. We further examined these terminals at the EM level and revealed onefold–twofold shrinkage in the sizes of vGluT1 labeled corticothalamic terminals in VP and RTN. Based on the experimental observations from the present study and our previous study [23] and the recent finding from others [19], we proposed a working model to elucidate the underlying cellular mechanisms may account for the alteration of thalamic circuitry in the mouse model of PVL.

### A working model of the vulnerability of corticothalamic synapses in the PVL mice

**Fig. 5:**
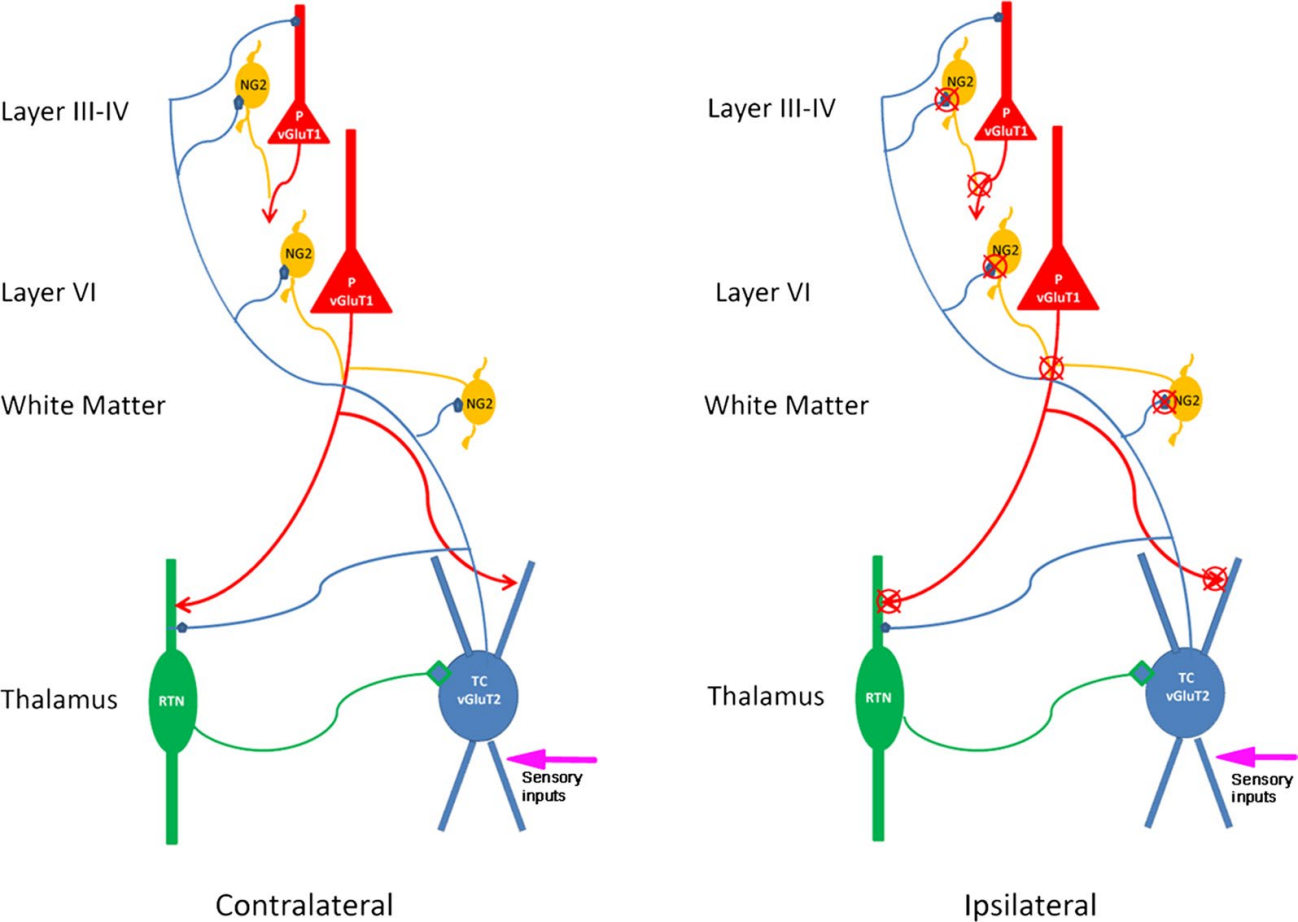
A working model illustrating the thalamocortical circuitry and the affected targets in the contralateral versus ipsilateral side of the brain in a mouse model of PVL. NG2 cells are represented in the white matter and in the cerebral cortex. The synaptic contacts between vGluT2 labeled terminals from thalamocortical (TC) cells (termed “TC vGluT2” cells) are formed in the developing white matter and the cerebral cortex. These synapses are altered in PVL mice, which may in turn affect the myelination of layer VI pyramidal (P) cells (termed “P vGluT1” cells) that may then change vGluT1 expression in their axon terminals in the VP and RTN (indicated by *red crosses*)

In this working model as shown in Fig. 5 , at the normal condition, as illustrated in the contralateral side, thalamocortical (TC) relay cells which express vGluT2 [9] (TC vGluT2) in the thalamic nucleus VP send out their axons through white matter, and they also send branches to synapse with GABAergic RTN cells. During postnatal development, these TC cell axons form synaptic contacts on NG2 cells in the white matter [23], and they also project to other NG2 cells in layer IV and layer VI and synapse on layer IV cells as well [19]. Layer III-IV cells and layer VI pyramidal cells which express vGluT1 receive TC synaptic inputs and send out their axons through white matter and form synapses on RTN cells and TC cells. GAB Aergic cells in RTN project back to TC cells, serving as a negative feedback loop. Note that axon bundles from vGluT1 containing layer VI corticothalamic cells are myelinated.In the ipsilateral side of PVL mice, vGluT2 positive thalamocortical relay cells are profoundly compromised, their terminals that synapse on NG2 cells in the white matter undergo structural, biochemical and functional changes, and vGluT2 expression is altered, resulting in the defects in both NG2 progenitor cell function and myelination of corticothalamic axons. Thalamocortical synapses on NG2 cells in layer III-IV and layer VI are also affected, ultimately leading to the change of vGluT1 expression in layer III-IV and layer VI cells, down-regulation of vGluT1 expression in corticothalamic terminals, and malfunction of the synaptic transmission.

### vGluT1 as a reliable synaptic marker for corticothalamic synapses

Synaptic plasticity depends, at least in part, on presynaptic mechanisms that have been associated with the probability of neurotransmitter release [22]. The amount of glutamate contained within synaptic vesicles and available for release is regulated by vesicular glutamate transporters (vGluTs) located on the membrane of the vesicles. Two of the three known vGluT isoforms, vGluT1 and vGluT2, are relatively abundant throughout the central nervous system (CNS), where they are expressed by specific glutamatergic neuronal populations and, with significant exceptions, do not co-localize in the same synaptic terminals [6, 13]. It has been suggested that vGluT1 is primarily found at synapses characterized by low-release probability and a capacity for long-term potentiation (LTP), whereas vGluT2 is primarily found at synapses characterized by high-release probability and a capacity for long-term depression (LTD) [5]. Our recent study also confirmed previous findings that the majority of thalamocortical terminals contain vGluT2 and the cortico-thalamic terminals derived from layer VI pyramidal cells contain vGluT1 [9, 14, 19]. In somatosensory thalamus, another source of similar terminals to corticothalamic synapses is derived from brainstem which mainly cholinergic and monoaminergic, but a large amount of these terminals do not form classical synapses [12, 16], therefore, vGluT1 labeled terminals here should be considered exclusively corticothalamic. In the present study, a quantitative analysis on vGluT1 immunostaining at the light microscopic level revealed a consistent reduction in labeled profiles in the somatosensory thalamus of the ipsilateral side of PVL mice. Although the degree of changes in three cases shows some variabilities, the trend is consistent. Such fluctuation could be related to the brain regions impacted by PVL. The reduction in labeled profiles in PVL mice was further confirmed by EM studies, showing decreased sizes of vGluT1 labeled terminals, which exhibited typical corticothalamic synaptic features in previous studies [12, 17]. The reduction in vGluT1 labeled terminal numbers could result from a down-regulation of vGluT1 protein due to the changes in layer VI pyramidal cells and also the alterations in axons and their terminals because of de-myelination. It is obvious that corticothalamic axon terminals were severely damaged, given the drastic ultrastructural changes. This target specific alteration can be reliably detected using vGluT1 labeling. Further investigation to explore the potential changes occur in cortical projection cells and possible associated changes in myelinated axons will shed light on the underlying thalamocortical circuitry which is highly implicated in PVL and other newborn neurological injuries or related diseases.

### Interaction of vGluT1 and vGluT2 in the thalamocortical circuitry in PVL

vGluT1 and vGluT2 are expressed in distinctive neuronal subtypes and involved in different functional systems. vGluT2 is primarily restricted to projections between and within subcortical areas, as well as thalamocortical projections, while vGluT1 is reserved for intercortical and corticothalamic projections [2, 8, 10, 13, 14, 23]. The pathology in corticothalamic terminals in PVL suggests that different vGluT proteins may be engaged in specific functional pathways. How is the corticothalamic pathway affected in PVL mice? We need to consider the thalamocortical circuitry as a whole in order to identify which may be altered at PVL condition. Our previous study [23] demonstrated that the postsynaptic targets of vGluT2 synapses were affected in the white matter of the ipsilateral side of PVL mice, the shrinkage of the postsynaptic profiles may be derived from NG2-expressing oligodendrocyte precursor cells (OPC). A recent study [19] using elegant transgenic techniques combined electrophysiological recording also demonstrated that vGluT2 labeled thalamocortical synapses specifically target NG2 cells in layer IV and layer VI and other neurons in these layers and provided strong evidence indicating that thalamocortical synapses are vGluT2 specific and are modified by somatosensory afferents [18]. Based on these findings, it is anticipated that the injury in PVL may firstly strike the thalamocortical relay cells which receive strong sensory inputs and form synaptic contacts with NG2 cells in the white matter and other cortical layers during early postnatal development (P2–P10). The alteration in synaptic transmission between vGluT2 expressing thalamocortical terminals and NG2 cells may compromise the differentiation and migration of these NG2 cells and ultimately affect the myelination of functionally organized axon bundles in the white matter and cerebral cortex and result in malfunction of the thalamocortical circuitry [18]. Furthermore, the impact of PVL injury may also affect the layer VI corticothalamic projecting pyramidal cells which express vGluT1 and thus down regulate the protein expression in the corticothalamic terminals and further alter the synaptic transmissions and their ultrastructure, the consequence of the impact would be the de-synchronization of the whole circuitry. The proposed working model presents a new pathway for elucidating the cellular mechanisms responsible for PVL injury. vGluT proteins are involved in regulating glutamate release in presynaptic terminals, the impact of the protein defection has a profound effect on glutamatergic transmission and thus alters the neuronal circuitry, such as thalamocortical pathway which control the conciseness level and cognitive functions of the animals. Future investigation to further understand the underlying pathology of PVL will be crucial for developing new treatments for this devastating human disorder.
